# Entropic Competition between Supercoiled and Torsionally Relaxed Chromatin Fibers Drives Loop Extrusion through Pseudo-Topologically Bound Cohesin

**DOI:** 10.3390/biology10020130

**Published:** 2021-02-07

**Authors:** Renáta Rusková, Dušan Račko

**Affiliations:** 1 Polymer Institute, Slovak Academy of Sciences, Dúbravská cesta 3, 84541 Bratislava, Slovakia; renata.ruskova@savba.sk; 2 Department of Plastics, Rubber and Fibres (IPM FCFT), Faculty of Chemical and Food Technology, Slovak University of Technology, 81237 Bratislava, Slovakia

**Keywords:** DNA, chromatin, polymer, molecular dynamic, coarse-grained simulations, supercoiling, loop extrusion

## Abstract

**Simple Summary:**

Chromatin dynamics and chromatin structure are a two-way relationship governed by polymer physics and active biological processes. Thanks to the research in the field of computational biology and modeling, computer simulations became indispensable in studying these complex relationships. It is now generally accepted that looped structures occurring in the intermediate range of ordering of chromatin are formed by a loop extrusion mechanism involving specialized proteins (structural maintenance complexes or SMCs). Although the motor activity of SMCs has been speculated for a long time, the motor activity of cohesin was discovered only recently (Davidson 2019). While evidence of the cohesin’s motor activity is missing, other mechanisms that could efficiently drive the loop extrusion without motor activity of SMCs have been discovered by computer simulations. These mechanisms account for transcriptionally driven loop extrusion or entropically driven loop extrusion by osmotic pressure. In our previous model, we have shown that the cohesin in handcuffed conformation can be pushed mechanically by emerging plectoneme formed during transcription, exerting pressure on the joint section of handcuffs. In the current work, we use coarse-grained molecular simulation to further explore the extrusion driven by supercoiling while employing much lower levels of supercoiling. Moreover, recent works favor non-topological binding of cohesin on fibers, which would solve a range of topological problems while bypassing other molecular machinery sitting on DNA. We show by means of computer simulations that supercoiling can drive loop extrusion without taking advantage of mechanic push on the joint section of cohesin handcuffs. As such, the work addresses current problems in molecular biology and employs advanced methods and original solutions in the study.

**Abstract:**

We propose a model for cohesin-mediated loop extrusion, where the loop extrusion is driven entropically by the energy difference between supercoiled and torsionally relaxed chromatin fibers. Different levels of negative supercoiling are controlled by varying imposed friction between the cohesin ring and the chromatin fiber. The speed of generation of negative supercoiling by RNA polymerase associated with TOP1 is kept constant and corresponds to 10 rotations per second. The model was tested by coarse-grained molecular simulations for a wide range of frictions between 2 to 200 folds of that of generic fiber and the surrounding medium. The higher friction allowed for the accumulation of higher levels of supercoiling, while the resulting extrusion rate also increased. The obtained extrusion rates for the given range of investigated frictions were between 1 and 10 kbps, but also a saturation of the rate at high frictions was observed. The calculated contact maps indicate a qualitative improvement obtained at lower levels of supercoiling. The fits of mathematical equations qualitatively reproduce the loop sizes and levels of supercoiling obtained from simulations and support the proposed mechanism of entropically driven extrusion. The cohesin ring is bound on the fibers pseudo-topologically, and the model suggests that the topological binding is not necessary.

## 1. Introduction

DNA is a highly structurally organized bio-polymer allowing for the high level of compaction encountered in living cells [1]. Starting from its double-helical structure that prevents the molecule from freely rotating about its axis, it further wraps around proteins, creating DNA–protein complexes called nucleosomes [2]. Nucleosomes are the basic building blocks of chromatin. As result, chromatin is seen more accurately as a fiber than a molecule. The number of nucleosomes per unit length of DNA varies from organism to organism [3]. The nucleosomes are separated by linker DNA, and the size of the linker DNA determines the biophysical properties of the chromatin fiber in terms of torsional stiffness [4].

Furthermore, at higher levels of organization the chromatin fibers form loops. The loops were first observed by the end of the 1970s on the dissolved metaphase chromosomes that showed such loops of about 100 kb attached to protein scaffold [5]. A first generalized mechanism for the formation of such loops was proposed in the early 1980s, while the existence of specialized protein complexes involved in the loop formation was proposed [6]. Since the 1990s, we have known of three proteins known as structural maintenance complexes, or SMCs, capable of such action, namely condensin I and II and cohesin [7]. The existence of the loops in interphase chromosomes was confirmed by a method from chromosome conformation capture methods, Hi-C, in the early 2010s [8,9,10]. The observed regions of increased contact probability were further named as topologically associating domains (TADs) probably due to similarity to the topological domains whose existence was long known in bacterial chromosomes. Today, the role of SMCs in loop formation and chromosome organization in organisms from bacteria to humans is generally accepted [11].

Although direct proof of the motor-like activity of SMCs is pending, it has been speculated for a long time, and the first models were proposed by Marko et al. in 2012 [12]. Evidence for a motor activity of condensin was indicated by multiple experiments. Nonetheless, the first direct proof was finally provided by Dekker et al. by using video recording of fluorescence microscopy experiments, which showed asymmetric loop extrusion by condensins [13]. Motor activity of cohesin is a more complicated story, and only recent papers show a weak motor activity in deproteinated DNA and in the presence of protein loader Nipbl in in vitro experiments [14]. Over time, several proposed mechanisms for protein-mediated loop extrusion have appeared, in which proteins may act as active motors out of which only a few have been modeled and simulated by coarse-grained simulations [15]. In other mechanisms, the existing models propose a motor-less role of the proteins and the process of extrusion driven by transcription [16] or by osmotic pressure [17,18].

In the case of transcriptionally driven loop extrusion, we proposed a model where a supercoiling generated during transcription accumulates from one side of the cohesin rings [16], which was one of the favored pictures of entrapment of chromatin fibers by cohesin [19]. The cohesin rings in the model embraced the fibers topologically in the form of handcuffs. The arising supercoiling pushes on the joint section of the handcuffs, moving the rings and extruding the loop. The model was proven to be successful in simulating symmetric as well as asymmetric loop extrusion, and it was fast enough to simulate extrusion at biologically relevant speeds reaching kilo base-pairs per second. At the same time, it solved a problem with directionality of the movement that naturally moves along the gradient of supercoiling, from the source of supercoiling inside the loop towards the borders of the domain. The transcription is also a process naturally occurring in the cells, hence offering an elegant explanation for the driving force behind the loop extrusion. The model for transcriptionally driven loop extrusion, considered by us earlier [20], involved direct pushing/squeezing of cohesin rings by chromatin fibers that were drawn together by transcription induced supercoiling. In our earlier work [20], we considered also the situations where the entire supercoiled loop needed to be reorganized since the progression of one cohesin ring was blocked by its contact with CTCF proteins sitting at the base of formed loops [20]. Here, considering the model where entropic competition between supercoiled and torsionally relaxed chromatin fibers results in loop extrusion, we focused on a situation where a relatively simple loop-forming TAD is generated. Cohesin rings are known to load near a promoter of an active gene [21] and after the transcription starts, the cohesin rings separate the region where the supercoiling is generated from the region where supercoiling can be relaxed by the action of DNA topoisomerases that are associated with CTCF at borders of TADs [22]. In our current model, we focus on considering the simplest situation with one transcribing RNA polymerase progressing with a constant speed and do not treat such interesting questions as what would happen if we had two converging or diverging polymerases.

In scope of the discoveries during the last two years, our computational model for transcriptionally driven loop extrusion would need to be reconciled with recent experimental findings. First of all, the supercoiling encountered in our previous simulations eventually became very strong, which has not been yet observed experimentally in human chromatin; moreover, such a dense supercoiling would lead to observation of anti-diagonal features on contact maps, which is not desirable. Secondly and perhaps more importantly, the works by Davidson et al. [14] and Kim et al. [23] showed that the cohesin is bound to the fibers at most pseudo-topologically or non-topologically, and hence the transcriptionally driven loop extrusion should not take advantage of pushing on the joint section of the cohesin handcuffs. The non-topological binding also provides the easiest solution for traversals of proteins or SMCs avoiding molecular machinery observed recently by experiments [24].

The aim of the paper is thus to show whether the model for transcriptionally driven loop extrusion can withstand the trial of time in the light of new experimental findings and if the transcription can still drive loop extrusion at lower levels of supercoiling and when the cohesin is loaded on the fibers in a non-topological manner. Last but not least, we show that transcriptionally driven extrusion in such settings is still capable of controlled loop extrusion at biologically relevant times, and its mechanism can be interpreted by finding similarities with the entropic models of loop extrusion.

## 2. Materials and Methods

We performed coarse-grained molecular dynamics simulations in Extensible Simulation Package for Research on Soft Matter [25,26]. The model consists of several key components. First of all, we used a circular beaded chain with torsional stiffness to represent a portion of chromatin fiber to be extruded. The reason for using a circular chain to represent chromatin fiber was to eliminate the size effects and effects of polymer ends. One bead of the chromatin fiber corresponds to *σ*_LJ_ = 10 nm containing 400 bp of DNA wrapped around two nucleosomes [27] and ~70 bps of linker DNA. The total size of our beaded chain was 150 beads, which represents a smaller loop of 60 kbp. The beads were bound by a strong harmonic potential, and a fully repulsive interaction potential in order to model excluded volume was installed. Furthermore, we imposed a bending stiffness *K*_b_ = 5, giving the fiber persistence length of 50 nm [28]. A classical beaded chain model does not have torsional stiffness. In order to include the torsional stiffness into the model, we included additional virtual beads that did not possess excluded volume interaction and exhibited only hydrodynamic drag *γ* with surrounding media. These additional beads are attached periaxially with respect to the main axis of the chromatin chain and their set to *γ* = *γ*_R_ = 1. Subsequently, these periaxial beads are interlocked by the torsional potential that creates an energy penalty for torsional deformation. Parameters and detailed procedures for how we build polymer models with torsional stiffness can be found in our earlier work [29,30,31]. 

Furthermore, in order to model the cohesin ring, we used a smaller beaded circle threaded on the circular chromatin chain in a pseudo-topological manner, embracing both fibers with a single ring. The ring consisted of 14 beads, each representing 10 nm. This made the maximum size of the protein in a rod-like configuration about 50 nm long, with the size of the opening in state threaded on the fibers between the experimentally reported 10 and 20 nm [32]. In order to simulate friction between cohesin and chromatin fiber, the hydrodynamic drag *γ*(*x*_c_) = *γ*_c_ of the virtual periaxial beads and adjacent real bead was increased for a particular bead found in the inner cross-section of the ring, defined as *x*_c_, along the simulated fiber. In the simulations, we investigated the effect of increased drag *γ*_c_ on the levels of accumulated supercoiling and speed of loop extrusion, while we used values of *γ*_c_ to be between 2 and 200 times larger than the drag of real beads *γ*_R_ = 1. In order to load the ring on the fiber, we used a modified approach described by Bonato et al. [33]. The ring was initially positioned around a loading bead (*x*_c_ = 0) in a folded handcuff conformation with two rings of 7 beads wrapped around the fiber. In the next step, the bending stiffness of cohesin was reinstalled, which caused the opening of the folded handcuff configuration. In addition to these initial steps, the bonds between beads that created the joint section of the handcuffs configuration were removed. At the same time, excluded volume interaction between cohesin ring and loading bead was increased to 3 *σ*_b_ (while maintaining excluded volume between beads of the fiber at 1 *σ*_b_), in order to prevent the pseudo-topologically threaded ring from sliding off from the chain. The increased size of the excluded volume may represent an increased body of RNA polymerase+TOP1 motor, which starts introducing supercoiling behind the ring just after its loading on the fiber. The summary of the bead setup with parameters and model equations of the interactions employed in our coarse-grained model is provided in Table S1.

Another advanced feature in our model as compared to classical beaded chain models is a motor representing an active bio-molecular machine that introduces negative supercoiling. Conceptually, this motor consists of an RNA polymerase associated with TOP1 topoisomerase that removes positive supercoiling, leaving flux of only negative supercoiling into the system [34]. In previous works, we showed implementations of both motors, introducing negative supercoiling with constant speed as well as that with constant force [30]. In the present model, we use a motor introducing negative supercoiling with a constant speed of 10 rotations per second. 

A new feature implemented in our model is moving nicks that allow releasing of supercoiling. These are placed one bead ahead of the moving cohesin rings. We have implemented this feature in order to eliminate the size effects of the simulations and to incorporate an assumption that the supercoiling outside the loop relaxes very quickly at TAD borders by TopIIb [22].

In order to calibrate time units of our simulations, we used a similar approach to the one in our work on supercoiling as a driving force behind postreplicative unknotting of DNA [35]. We first performed a series of simulations in order to find the speed of rotations of the simulated motor where *γ*_c_ = *γ*_R_ = 1, i.e., where there is no excessive friction between cohesin and the fiber, and no accumulation of supercoiling was observed. We found such conditions when our motor turned once per 36,000 integrations. Subsequently, we performed very long simulations and no visual writhing or evident pushing of cohesin ring was observed over a very long simulation run. Our integration step was set to ∆τ = 0.0025 time units, and one full rotation took 90 time units. Next, we determined the relation between simulated time units and the physical time by the approach used by Di Stefano et al. such that the time unit corresponds to Stokes’ time τ = 6πησ^3^/*k*_B_*T* = 4.5 µs*η, where η is the viscosity of surrounding media [36]. In order to obtain 10 rotations per second, we considered that the viscosity of surrounding media is 240 times that of pure water. This value is reasonable, as the values of viscosity in the presence of molecular crowders in living cells were reported experimentally to be as high as 220,000 times that of pure water [37]. The longest simulations with *γ*_c_ = 2 *γ*_R_ took three weeks to simulate, and the shortest time, with runs of *γ*_c_ = 200 *γ*_R_, took about two days. Calibration by using increased viscosity helped to save computational time and made performing the simulations of the loop extrusion mediated by friction 240 times faster.

Additionally, we employed computational analytical tools to calculate writhe and twist in extruded loop developed previously [38].

## 3. Results and Discussion

### 3.1. The Rate of Loop Extrusion Is Controlled by the Friction Imposed by Cohesin

As we mentioned in the Introduction, there are several existing mechanisms currently considered to drive loop extrusion. It has been recently discovered that cohesin can exhibit a weak motor-like activity, actively extruding chromatin fibers. This activity has been shown in in vitro experiments on deproteinated DNA in the presence of Nipbl loader [14]. This motor activity is relatively weak in terms of used energy but efficient in terms of extrusion rate of 2.1 kbps [14]. Besides the proven ability of cohesin to act as a motor, there were other different mechanisms proposed to efficiently extrude the fiber and as such able to enhance the extrusion [16,17,18]. These include purely diffusive extrusion of loops and transcriptionally driven extrusion. In our previous model for the transcriptionally driven extrusion, we showed that the accumulation of supercoiling generated during the transcription can be very efficient and powerful for extruding loops [18]. Our previous model, however, inherently expected handcuff conformation of cohesin rings and mechanical pushing of the emerging supercoiling on the joint section of the handcuffs. Therefore, we wanted to test whether the model with transcriptionally induced supercoiling could still enhance loop extrusion in modified conditions. In these conditions, the cohesin is represented by a single ring embracing both fibers, but still inducing friction between the ring and the fibers. The levels of the friction between the fibers and cohesin are intended to be used in order to control the levels of accumulated supercoiling and possibly the rate of loop extrusion. Although the levels of supercoiling can be controlled by changing the speed of the motor, the speed of generation of supercoiling is a biologically fixed parameter of around 10 rotations per second generated by RNA polymerase [39]. Therefore, we assume that varying the speed of the motor generating the supercoiling would not be correct. We consider varying the friction between cohesin and the fibers as the best option. The experimental work by Stigler et al. indicated that this friction can be very high, such that self-diffusion of the cohesin without the help of an active process would take too long for efficient loop extrusion in biologically reasonable times [40]. On the other hand, the friction between cohesin and fibers not only would alter the release of the supercoiling by affecting axial rotations of the fiber but will also impose higher drag on the mutual translational movements of cohesin and fibers, i.e., diffusion of the rings. This makes the relation between the friction, accumulation of supercoiling and diffusion of cohesins rather non-trivial and needs to be explored by the simulations.

**Figure 1:**
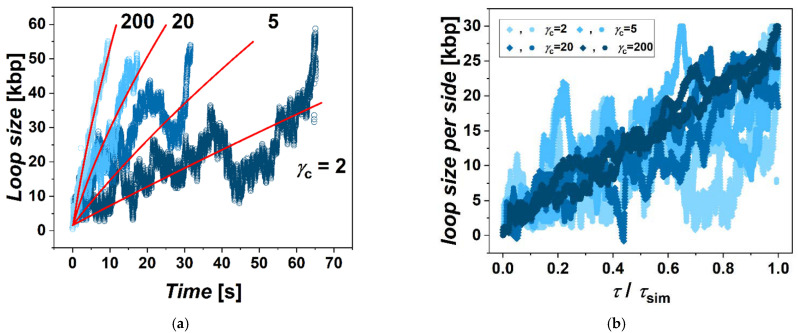
The simulations of loop extrusion. (**a**) The loop sizes are shown as a dependence of time for four settings of friction between cohesin ring and chromatin fiber *γ*_c_ = 2, 5, 20 and 200. Graphs indicate that the rate of loop extrusion increases, with larger friction induced at the position of the ring. The red lines show the fits obtained by numerical solving of the system of differential equations proposed to describe the process of entropically driven extrusion (Equations (1) and (2)). (**b**) The progress of loop extrusion for individual arms of the loop in terms of their sizes is calculated as a difference of the position of the ring on the arm and the position of the motor. The graph shows that the extrusion becomes more stochastic for lower settings of *γ*_c_s, making the extrusion also more asymmetric.

**Figure 3:**
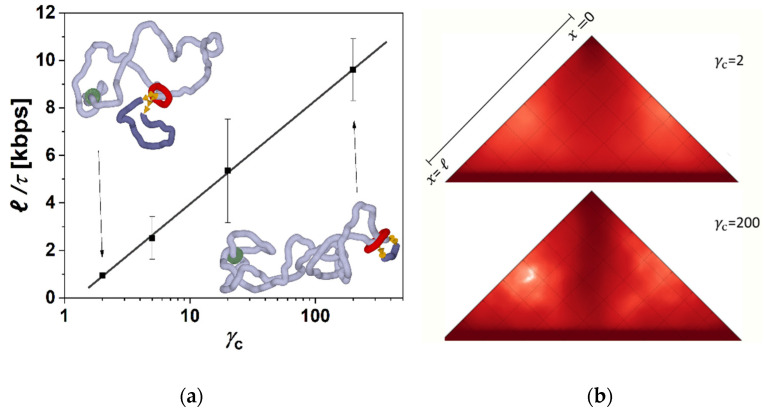
The higher friction between cohesin ring and fiber induces higher levels of supercoiling and extrudes loops at higher rates. (**a**) Loop extrusion rate for different settings of *γ*_c_ imposing friction between cohesin ring and chromatin fiber, resulting in different levels of accumulated supercoiling. The loop extrusion is shown as a function of imposed friction between cohesin and fibers. The insets show snapshots from the end of the simulations at the lowest and highest settings of the friction. (**b**) The contact maps are calculated during the loop extrusion. The contact maps were calculated along the trajectories as the average over 10 simulation runs. The maps show the loss of ant-diagonal when the loop extrusion is more stochastic, i.e., random, when lower settings of friction between cohesin ring and chromatin fiber allow the fiber to sample larger conformational space. The data acquisition for the contact maps calculation stopped once the ring slipped away from the chromatin fiber. Biologically, the rings should stay stuck to CTCF’s proteins at borders of the domain [56], which would emphasize the loss of the anti-diagonal in simulations with lower friction.

This determined the first step in the simulations, when after constructing the model, we performed series of simulations with different frictions between cohesin and fibers imposed. These simulations showed a clear effect and systematic dependence in terms of the extrusion rate as a function of the friction (Figure 1a). First of all, we observe that the loops are extruded also by using a single ring embracing both fibers in so-called pseudo-topological binding [14]. In the next step, we evaluated the sizes of the loops with the simulation time. The calculated loop size *ℓ* with respect to the simulation time resulted in the loop extrusion rate that we show later in the discussion of the results (Figure 3). From the simulations, we observe that the loops are directionally extruded with a persistent motion of the ring in the direction from the motor towards the opposite side of the domain represented by the circular beaded chain to the point when the cohesin ring slips away from the beaded chain. This directional movement prevails even for very low friction imposed at the level of *γ*_c_ = 2. In the case of very high friction between cohesin and the fiber, the movement is even more straightforward, with much less fluctuation of the instantaneous values of loop size (Figure 1b). The extrusion also becomes very symmetric. The time of extrusion of the whole loop in the case of the highest *γ*_c_ in the simulation was 15 times faster than the lowest *γ*_c_ = 2. In the case of employing low gammas, the extrusion becomes more stochastic and asymmetric. The simulations also indicate that the dependence of the loop extrusion rate on the friction saturates when the increase of *γ*_c_ from 2 to 20 speeds up the extrusion 4 times, but a further increase of *γ*_c_ by a factor of 10 to *γ*_c_ = 200 speeds extrusion only 2 times. This indicates that there exists an optimum value of friction mediating the loop extrusion, after which the loop extrusion rate reaches its maximum, and later, at much higher frictions, the extrusion would probably decrease or even stop. In our simulations, however, we do not explore frictions that high as this would require decreasing integration steps and would make the simulations unfeasible in terms of the computational time.

**Figure 2:**
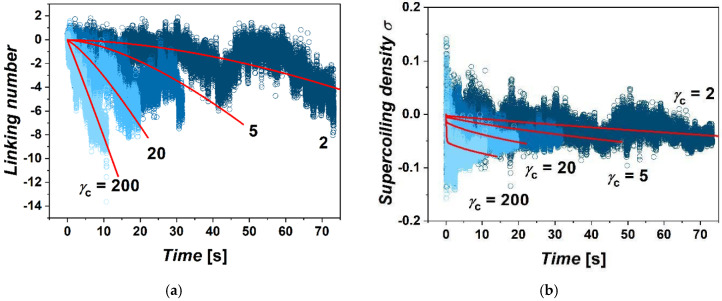
The simulations of supercoiling. (**a**) The graphs show accumulations of linking numbers in terms of White’s formula ∆*Lk* = ∆*Tw* + ∆*Wr*. The accumulation of the linking number is shown as dependence of time for four settings of friction between cohesin ring and chromatin fiber *γ*_c_ = 2, 5, 20 and 200. (**b**) Density of supercoiling obtained as *σ* = ∆*Lk*/*ℓ*. The red lines are the fits obtained from solving the differential system of equations describing the accumulation of supercoiling with a moving permeable boundary defined by the position of cohesin.

Differences are also found in the accumulation of supercoiling within the extruded loop as a function of imposed friction. We evaluated supercoiling within the extruded loop in terms of linking number and density of supercoiling (Figure 2). For this, we computed values of the writhe and twist within the extruded loop bordered by the position *χ*_c_ of the cohesin on the chromatin fiber for each frame of the simulation. The sum of the calculated writhe and twist gives the value of linking number ∆*Lk* according to Fuller’s theorem [41]. This can be used to calculate the density of supercoiling as *σ* = (*Lk* − *Lk*_0_) / *Lk*_0_ = ∆*Lk*/*Lk*_0_, where *Lk*_0_ is the linking number of the relaxed state, and here, it will correspond to the number of turns of relaxed DNA *Lk*_0_ = ~40 turns per bead [42]. During extrusion of the loop, the supercoiling inside the loop is relaxed by the influx of relaxed portions of the fiber into the loop, but also by escaping of supercoiling through the ring by axial rotation of fibers. During the simulations, from several tens to hundreds of rotations of the motor are performed. This is consistent with the size of the loop, which is 60 kbp; hence, the extrusion of the whole fiber should take time in order of 10s of seconds. The total number of rotations of polymerase introducing supercoiling at a rate of 10 rotations per second should be also in the order of hundreds. The calculated linking numbers indicate that in the case of large gammas, about 87.5% of rotations are relaxed. In the case of simulations with low *γ*_c_s, the loop loses above 99% of rotations both due to axial rotations as well as by dissolving the supercoiling in the un-supercoiled portions of chromatin flowing through the cohesin ring into the loop. The fluctuations of the linking number in terms of its amplitude originate mostly from the fluctuation of the twist. The overall fluctuations are more intense in the case of the systems with lower settings of *γ*_c_. In the case of lower friction, the accumulation of supercoiling has a strong non-equilibrium character. On the other hand, the accumulation of supercoiling in the case of larger friction strongly limits the effusion of supercoiling by axial rotations, while the gradient of supercoiling between the motor and position of cohesin quickly re-establishes, yielding the linear dependence of ∆*Lk* with time.

### 3.2. Mathematic Model of Transcriptionally Driven Loop Extrusion

In order to understand and explain the mechanism of the extrusion seen in the simulations, one may think of entropic competition of supercoiled and torsionally relaxed portions of the fiber. A similar problem has been studied by Orlandini et al. for two competing knots on a circular polymer chain separated by a sliplink [43]. In their paper, the authors showed that increasing the topological complexity of the knotted part decreases the polymer’s conformational space. Consequently, the part of the molecule with a more complex knot tries to increase its entropy by pulling a larger portion of polymer through the border separated by the sliplink. Hence, in an analogical picture, one could think of the compacted supercoiled loop trying to restore its entropy by pulling the relaxed portion of the fiber through the cohesin ring. However, the model for the competition between the supercoiled and torsionally relaxed portions of fiber poses more difficulties for simulations. This is because, unlike the problem of Orlandini et al., our system is not topologically constrained. The supercoiling can escape by axial rotations. Even if we limit the axial rotations by friction, the diffusive motions of cohesin can still effectively erase the supercoiling from the extruded loop. Therefore, one needs to think of a more complex kinetic equilibrium. In the case of the kinetic equilibrium, the supercoiling is continuously introduced by the motor. In the model, the intrinsic minimum energy point would be when the cohesins are sitting just at the position of the polymerase. However, this is not possible due to the increase in excluded volume between the bead representing polymerase and the cohesin ring. Hence, the only way for the system to decrease its energy is by moving the cohesin ring ahead from the transcription site, which induces relaxed portions of the fiber into the loop. This causes a temporary drop in energy; however, the supercoiling is soon replenished; hence, the cohesin moves again to attain a new energy minimum state (Video S1). In this way, the loop is extruded by cohesin movements, which follow the minimum energy path. 

For a mathematical description of this picture one would need to describe the non-equilibrium evolution of supercoiling along the fiber. For this purpose, we use Fick’s second law of non-equilibrium diffusion and balancing the supercoiling along the fiber as suggested by Brackley et al. in their stochastic model for supercoiling [44]:

(1) $$\frac{\partial \sigma \left(x,\tau \right)}{\partial \tau }\;={\;D}_{\sigma }\left(x\right)\frac{\partial^{2}\sigma \left(x,\tau \right)}{\partial x^{2}}$$

where *σ* denotes the density of supercoiling along the fiber at the distance *x*, represented by the position of the bead on the simulated chain and at the simulated time *τ*. Although the diffusivity of supercoiling is denoted here as a variable of the position $D_{\sigma }\left(x\right)$, its value in terms of *γ*_R_ imposed on the periaxial beads in the simulations along the fiber is the same, and set to *γ*_R_ = 1 for all *x* except the position of cohesin *x*_c_. The value is increased at the position of cohesin *x*_c_, such that *D*_c_ = $D_{\sigma }\left(x_{c}\right)$ = *k*_B_*T*/*γ*_c_ = $D_{\sigma }$*γ*_R_/*γ*_c_ with *ε* = *k*_B_*T* = 1. Initially, at the time *τ* = 0, the fiber is torsionally relaxed $\sigma \left(x,\tau \right)\;=\;0$. For times *τ* > 0, the supercoiling is continuously introduced at the position of *x* = 0, which is mathematically treated as a setting of the boundary condition ${\left(\partial \sigma /\;\partial t\right)}_{x\;=\;0}\;=\;{\dot{\sigma }}_{P}+D_{\sigma }\left(\partial^{2}\sigma /\partial x^{2}\right)$, where the speed of production is given as ${\dot{\sigma }}_{P}\;=\;$10 rotations per second. The equation is solved within the region *x* ϵ <0, *x*_c_ >, which represents the size of the loop. Because the movement of cohesin is one of the most prominent features of our proposed model, this needs to be included in the solution. Hence, we need to describe the movement of cohesin and solve the diffusion of supercoiling as a problem with moving boundary set as ${\left(\partial \sigma /\;\partial t\right)}_{x\;=\;x_{c}}\;=\;D_{\sigma }\left(\partial \sigma /\partial x\right)-D_{\sigma }\left(x_{c}\right)\left(\partial \sigma /\partial x\right)$ [45]. The movement of cohesin can be described by the equation for displacement from the overdamped Langevin equation, where we neglect the noisy part of the equation.

(2) $$\frac{\partial x_{c}}{\partial \tau }\;=\;-\frac{1}{\gamma_{c}}\left(\frac{\partial u}{\partial x_{c}}\right)$$

The term on the right side of the equation originally describes the change of energy potential for a particle moving in a field. The *γ*_c_ reflects the dissipation of the energy during the movement. It should be noted that the value of friction between the fiber and the rings is not the result of simulations but is introduced as a parameter setting into the model. Simulating the friction starting from first principles would require the employment of a fully atomistic model, and it would encounter several issues starting from the computational complexity of the problem [46]. The atomistic computer simulations of chromatin fiber with a comparable size to ours were undertaken recently, while the system of 83 kbp consisted of one billion atoms [47]. However, such simulations were only possible thanks to the employment of 100 thousand CPUs, while the time scales of seconds or minutes of real time were far beyond the reach of the atomistic simulations, as we could not take advantage of coarse-grained time units. If one was able to undertake the simulations of this kind of magnitude though, the friction coefficient could be explored as a measured property dependent also on cohesin ring mass, its hydrodynamic drag, etc., which would be then a part of Equation (2). Instead, in our simulations, we explore the behavior of supercoiling and loop extrusion rate for a wide range of frictions, which is a parameter setting. Hence, the rings themselves could be even fixed, and we would still obtain the same simulation for a given setting of friction. The wide range of frictions employed is plausible based on the experimental work of Stigler et al. [40], which suggested that friction can be very high. Physically, the friction arises from a combination of inter-surface adhesion, surface roughness and deformation. Based on the results reported by Stigler et al. [40], the molecular surface of chromatin appears to be very rugged for a diffusing cohesin due to the presence of individual nucleosomes, nucleosome arrays and other protein obstacles and machinery. In our model, we suppose a uniform distribution of the obstacles that are represented by a given averaged setting of the friction parameter *γ*_c_. In principle, however, the beaded model also provides the possibility for implementing a site-specific friction within the resolution given by the particular level of coarse-graining employed in the beaded model. This would be interesting, e.g., to investigate the effects of local modulation of friction due to chromatin folding and the presence of protein/DNA regulatory hubs such as those identified at specific sites on micro-C contact maps [48]. At the same time, we consider the right term of Equation (2) to be the change of internal energy associated with the drop in energy of supercoiling as new relaxed portions of fiber infuse into the loop once the cohesin advances in movement. In our mathematical model, we assume that the temporary drop of energy of supercoiling induced by cohesin movement propels the cohesin movement and loop extrusion. The energy of supercoiling can be expressed as *u* = *K*.∆*Lk*^2^, where the constant *K* represents energy of fiber’s force interactions such as bond stretching, angle bending and torsional flexibility [42]. 

The second-order differential equation describing non-equilibrium diffusion is notorious for having analytical solutions only for a limited group of problems [45]. Hence, the system of differential equations with the moving boundary proposed above needs to be solved numerically. The equations are solved and fitted over loop sizes and linking numbers obtained along the simulated trajectories. The parameters $D_{\sigma }$ and *K* were obtained by a concatenated fit over all trajectories with a particular value of *γ*_c_. The fitted dependencies are shown in Figure 1 and Figure 2, together with the values of loop size and linking number obtained by coarse-grained simulations. The values of model parameters are obtained per two fibers going through one ring. As our set of equations treats the problem in 1 dimension with one motor on one side and cohesin boundary on the other side, the coefficients obtained should be therefore halved in order to properly grasp the fact that effusion occurs through two fibers embraced by a single ring. As underlined in the paper by Bonato et al., using a similar approach to mathematical modeling in order to describe the movement of cohesin ring along the fiber, this aspect of the mathematical treatment does not qualitatively affect the results [33]. 

As for the obtained value of diffusivity of supercoiling, $D_{\sigma }$ = 1.6, agrees well with the value obtained by the study of plectoneme dynamics of braided polymers like DNA by Forte et al. [49]. In their study, the authors observed that the dynamics of twist is faster than that of writhe, while the obtained decoupled diffusivities were *D*_Tw_ = 2.0 and *D*_Wr_ = 0.1, giving the value of diffusivity of supercoiling as a weighted average with contributions of each based on whether the system accommodates a stable plectonemic or straight conformation. In our simulations, we also see the fluctuations of ∆*Lk* affected in greater part by the fluctuations of twist, implying that the dynamics of twist is much faster. Additionally, we note that the diffusivity of supercoiling in real units obtained from transforming *σ*^2^/*τ* will be much larger than the diffusivity of whole plectonemes observed by Dekker et al. to be *D* = 0.1 kbp2/s [50]. However, the diffusivity of supercoiling along the fiber agrees well with expected rotational mobility of DNA that was experimentally observed to be in order of thousands of rotations per second [51]. As observed from the simulations and supported also by mathematical modeling, in the case of large gammas, the equilibrium state of the gradient of supercoiling along the fiber is relatively quickly re-established after each movement of cohesin, as the high friction with cohesin limits losses of supercoiling (Video S2). As result, the value of the average supercoiling of the loop increases rather linearly over time. On the other hand, in the case of low gammas, the gradient of supercoiling along the fiber is more time-dependent as the semipermeable boundary formed by cohesin allows escaping of supercoiling by axial rotations to a much greater extent (Video S3). As result, the average value of ∆*Lk* starts to increase more intensely in later stages of the loop extrusion. This is consistent with the expectation that in an infinitely long loop, the average density of supercoiling would approach a ratio of turns introduced by polymerase versus the average loop extrusion rate. Additionally, we would like to stress, the purpose of the mathematical model presented in this section is not to provide a full quantitative treatment for describing precisely the loop extrusion in vivo by two parameters; rather, we aimed to provide an additional support in terms of qualitative agreement between the simulated data and fits from the mathematical model that would support the proposed mechanism of the transcriptionally driven loop extrusion mediated by friction between cohesin rings and chromatin fibers.

### 3.3. Towards Entropically Driven Loop Extrusion, Osmotic Pressure and Other Models

As for the value *K*, the energy constant of supercoiling, we obtained a value from the fits of *K*_sim_ = 3.4 × 10^3^. The halved value per arm is 1.7 × 10^3^, which is similar to the value of the energy constant given by Vologodskii as *K* = 1100, and it is referred to as a dimensionless constant [52]. On the other hand, it was pointed out that the dimension of the constant is probably per bp [42]. In our case, we use arbitrary units in terms of unitless beads on both sides of the equation; hence, a change of the units would not change the constant obtained from the fit. The difference of the fitted value of the energy constant is most likely given due to the internal settings of the force field in our model and can be improved in future works. In order to decouple contributions of other forces acting on the extrusion, one would need to go into a more detailed description of the energy term. For example, Bonato et al. have shown that chromatin stiffness and compaction play a crucial role in enhancing diffusive loop extrusion [33]. In their model and subsequent mathematical description, they proposed that the change of the loop’s internal energy follows the equation $u\left(x_{c}\right)/k_{B}T\;=\;8l_{p}/x_{c}^{2}+c\log\left(x_{c}\right)$, where the first term on the right side of the equation represents energy penalty due to the chromatin stiffness that would drop as the loop size grows, AND the second term represents the entropic cost of looping. When these terms are added to our energy term given by the energy of supercoiling *u* = *K*.∆*Lk*^2^ in (Equation (2)), the fitted value of *K* drops to 1550 (*c =* 0.03); however, the quality of the fit remains the same (Figure S1). In general, adding more terms into the functional decreases degrees of freedom and keeps the quality of fits the same if not improved. Additionally, one may think of other energy contributions acting in favor of the loop extrusion, such as opening the folded conformation of the cohesin rings in the early stages of the simulations. On one hand, incorporating the energy functional used by the diffusive model into our model provides a perspective for merging both models that both exhibit enhancement of the loop extrusion by entropic mechanism. On the other hand, providing a rigorous description of the energy term and decoupling energy contributions arising from bending, stretching, unfolding the cohesin, motor activity that acts as a constant speed, i.e., infinite energy motor, etc. is beyond the scope of the present paper. Moreover, the levels of coarse-graining in our work and in Bonato et al.’s work are currently different, which makes a direct comparison of the results complicated. Thus, we instead intend to show that the proposed mechanism is still able to drive loop extrusion at varying levels of friction, accumulated supercoiling and non-topologically bound cohesins, with proof-of-concept simulations supported by a mathematical description. As the energy term ∂*u*/∂*x* is a definition of chemical potential *μ*, the loop extrusion driven by the change of the chemical potential can be considered as an entropic process. The difference of the chemical potentials on the interface determined by the moving cohesin, *μ*(*σ*, *p* + Π) on supercoiled side and *μ*(*σ* = 0) on relaxed side, corresponds to the definition of osmotic pressure. Hence, one may think of an analogy of the osmotic process when the difference of concentrations on the interface drives the solvent into the concentrated phase. In our case, the solvent would be represented by relaxed portions of the fiber that “dissolve” the accumulated supercoiling within the extruded loop. The similar influx of chromatin fiber works in the mechanism of loop extrusion by an osmotic ratchet [53], where the fiber “dissolves” the concentration of cohesin rings loaded on the fiber.

### 3.4. Biological Contexts and Implications

As we mentioned earlier, the existence of loops and their formation by loop extrusion mechanism involving the presence of specialized proteins is nowadays generally accepted. Less consensus has been attained about the role of SMC proteins and mechanisms by which they can perform the extrusion. We know now that cohesin is capable of self-motional motor activity. This was shown to be relatively weak in terms of consumed ATP energy, ca. 1.7 ATP molecules per cohesin per second, but very effective in terms of extruding rate, 2 kbp [14]. On the other hand, before the motor activity of cohesin was experimentally shown, other biological and biophysical processes that can enhance loop extrusion were discovered by computer simulations [16,17,18]. In the case of transcriptionally driven loop extrusion, RNA polymerase is known to be one of the strongest biomolecular motors [54]. As we have shown earlier, negative supercoiling generated by RNAP+TOP1 complex can be a very effective driving force for the loop extrusion at high levels of supercoiling conserved in the loop [16]. In Section 3.1, we showed that even if much of the supercoiling is continuously relaxed to low levels by axial rotations, the energy of generated supercoiling still represents an effective force driving the loop extrusion. Figure 3a shows loop extrusion rates obtained as averages over five independent runs for each setting of friction between chromatin fiber and cohesin ring for the values of *γ*_c_ = 2, 5, 20 and 200 that were the main scenarios discussed in this paper. The loop extrusion rates are shown as a function of a product of the imposed friction. As the figure shows, the loop extrusion rate still increases with increasing friction as the energy of accumulated supercoiling increases, but it follows a logarithmic relation, showing the saturating effect on the loop extrusion rate. Biologically, the speeds should be such that the entire human genome could be extruded within minutes [14]. We may see that biologically relevant rates consistent with experimental measurements between 0.5–2 kbps [14] are achieved mainly for lower settings of *γ*_c_ ≤ 20. 

The corresponding average levels of accumulated supercoiling observed in the simulations in terms of linking number ∆*Lk* over the distance of 60 kbp range from −2 for the lowest friction *γ*_c_ = 2 to −10 for the largest friction *γ*_c_ = 200 employed. These values, however, represent the total value along the chain, while in reality, the supercoiling creates a gradient from the position from transcription site (TSS) *x* = 0 towards the site of release of supercoiling at the position of cohesin, *x* = *x*_c_. The site-specific linking number ∆*Lk* calculated at a given bead can be considered as the bead’s local density of the supercoiling *σ*. In this way, for *γ*_c_ = 200, the values measured and modeled at the position of the bead representing transcription site in our simulations reach *σ*(*x* = 0) = −12%. At the same time, the supercoiling drops towards the position of cohesin, where the value is maintained by the semipermeable boundary at *σ*(*x*_c_) = −5%. The evolution of ∆*Lk* along the chain and simulation time obtained from the mathematical model is given in Videos S2 and S3. The profile of ∆*Lk* obtained as average over the simulation run is given in Figure S2. The total value of *Lk* is the integral under the curve. In the case of low friction, the values range from *σ* = −7% at the TSS to *σ*(*x*_c_) = −0.2%, where the supercoiling drops more dramatically along the fiber, at the position of cohesin. The values of local supercoiling at the transcription site are a bit lower than predicted by the stochastic model of supercoiling-dependent transcription by Brackley et al. [44]. However, the values are in good agreement with the local DNA supercoiling determined for genes with low, medium and high expression around the position of TSS in terms of crosslinks (CLs) by psoralen photobinding in vivo by Kouzine et al. [55]. The faster decay of supercoiling in the simulations with lower *γ*_c_s is consistent with the profiles measured by Kouzine et al.

The lower levels of supercoiling also allow for higher conformational statistics and fluctuations of chromatin fiber than in the systems with strong supercoiling. The insets of Figure 3a show two corresponding snapshots from molecular simulations comparing the resulting structures with lower and higher levels of supercoiling. The higher conformational statistics within the extrusion are also reflected in the contact maps by the disappearance of the anti-diagonal feature that is prominent in the case of extrusion with strong supercoiling and which creates plectonemic conformation of the loop (Figure 3b). The contact maps on Figure 3b were obtained as averages from 10 trajectories. One has to note, that in our simulations the fiber does not contain CTCF proteins at the end of the domain, and the simulation stops after the cohesin reaches the end of the loop and unloads. In a more biological setting, the cohesin would stick to CTCFs and stay there while the simulation would continue for a period equivalent to 20 min of biological time [56]. This would enhance the appearance of the anti-diagonal feature on the simulated contact maps and emphasize the difference between the contact maps obtained for systems with low and high levels of supercoiling.

## 4. Conclusions

We have performed coarse-grained molecular dynamics simulations of supercoiled chromatin fiber with the cohesin ring mediating loop extrusion. The simulations showed that the levels of supercoiling can be controlled by the friction imposed between cohesin and chromatin fiber. The higher levels of supercoiling accumulate when imposing larger friction. At the same time, the rate of the loop extrusion increases, but it shows a saturation effect. The model also shows that supercoiling even at its low level represents an effective force to enhance the loop extrusion.

The model shows that the supercoiling can extrude the loop even if the ring is bound pseudo-topologically. During the pseudo-topological binding, the ring embraces both fibers and emerging supercoiling cannot take advantage of mechanic push on the joint section of cohesin handcuffs, like in our previous model. The loop extrusion is realized by following the minimum energy path, while the energy of supercoiling drops when new relaxed portions of chromatin fiber flow into the loop through the interface held by a cohesin ring. This means that the extrusion is driven by the change in chemical potential at the interface separating the supercoiled and non-supercoiled portions of the fiber. As the process is driven by the change in chemical potential, the loop extrusion driven by competition of supercoiled and torsionally relaxed fibers can be considered as an entropic process. The dissolution of supercoiling in new portions of fiber is analogical to the proposed entropically driven loop extrusion with osmotic pressure. The relaxed fiber represents solvent diffusing into the loop through the interface of the cohesin ring, trying to dissolve the accumulated supercoiling.

Based on the simulations and mathematical model, we conclude that the topological binding is not necessary as far as the cohesin keeps a thermodynamic coupling between cohesin and fiber. We assume the presence of the ring could be replaced by an abstraction of a special bond.

The mathematical modeling of the loop extrusion driven by the energy of supercoiling suggests compatibility of the models for loop extrusion driven by the energy of supercoiling and the model proposed for motorless extrusion by stiffness and compaction-enhanced diffusion. At the same time, one may think of our model as a mechanism that would enhance the loop extrusion along with the weak motor activity of cohesin or pushing through the osmotic ratchet.

## Data Availability

The data presented in this study are available on request from the corresponding author. The data are not publicly available due to the size of the MD trajectories.

## Supplementary Materials

The following are available online at https://www.mdpi.com/2079-7737/10/2/130/s1, Video S1: Visualization of molecular dynamics trajectory and simulated loop extrusion obtained for *γ*_c_ = 200; Video S2: Mathematical modeling of loop extrusion at high levels of supercoiling accumulated at high friction, *γ*_c_ = 200; Video S3: Mathematical modeling of loop extrusion at low levels of supercoiling accumulated at low friction *γ*_c_ = 2; Table S1: Model setup of bead interactions for chromatin fiber and cohesin; Figure S1: Fits of the system of mathematical Equations (1) and (2) over the simulated linking number and loop sizes when incorporating the energy terms for stiffness; Figure S2: Profile of supercoiling along the fiber obtained as average over the whole simulation.
