# Supplementary material for: Entropic Competition between Supercoiled and Torsionally Relaxed Chromatin Fibers Drives Loop Extrusion through Pseudo-Topologically Bound Cohesin
Source: Biology (Basel). 2021 Feb 7;10(2):130. doi: 10.3390/biology10020130 (PMC7915857; doi:10.3390/biology10020130)
Supplement: Supplementary file 1 [file biology-10-00130-s001.zip › biology-1043689-suppl-final.docx]

Supplementary Materials

Entropic Competition between Supercoiled and Torsionally Relaxed Chromatin Fibers Drives Loop Extrusion through Pseudo-Topologically Bound Cohesin

Renáta Rusková and Dušan Račko

Video Legends:

**Video S1**: Visualization of molecular dynamics trajectory and simulated loop extrusion obtained for γc = 200. The frames were rendered by using 3D coordinates obtained during the molecular simulations of a representative run. The green clutch represents transcription site consisting of RNA polymerase associate\d with TOP1 topoisomerase [1]. The red ring represents pseudo-topologically bound cohesin ring embracing both fibers. The hydrodynamic drag of peri-axial beads forming torsionally restrained fiber in our model is increased for the beads inside the cross section of the ring imposing the friction between cohesin ring and chromatin fiber. The portion of the loop that has been extruded is shown in light blue. Dark blue shows the portion of the chromatin fibre to be extruded. Yellow cones show the moving nicks that were implemented in order to simulate fast effusion of supercoiling outside the loop mediated by TobIIb topoisomerase [2].

**Video S2:** Mathematical modelling of loop extrusion at high levels of supercoiling accumulated at high friction, γc = 200. The video shows evolution of the profile of supercoiling along the simulated chromatin fibre. The supercoiling is evaluated in terms of excess of linking number introduced by the action of polymerase associated with TOP1. The topoisomerase TOP1 sits behind the polymerase and removes primarily the positive supercoiling, leading to accumulation of negative supercoiling. The excess of linking number ∆*Lk* at given bead gives the site-specific density of supercoiling over a genomic distance corresponding to the coarse grained size of the bead, i.e., 400 bp. The profile shows a decay along the fibre towards the position of the cohesin ring. The cohesin ring creates a semipermeable boundary allowing a partial torsional relaxation by axial rotations. In the current setting of the imposed friction the supercoiling accumulated at the borders of the boundary rises to −5 %. The level of supercoiling at the site of transcription keeps growing and reaches −12%. The model qualitatively agrees with the experimental measurements of supercoiling by means of psoralen photobinding reported earlier [3]. Quantitatively, the levels would reach the levels reported for the sites with medium and high levels of transcription.

**Video S3:** Mathematical modelling of loop extrusion at low levels of supercoiling accumulated at high friction, γc = 2. The video shows evolution of the profile of supercoiling along the simulated chromatin fibre. See above—caption to Video S2. The level of supercoiling at the site of transcription keeps growing and reaches −7%. The accumulation of supercoiling at the boundary is very low ~0.2%, as the low levels of friction imposed between the fiber and the ring are not able to prevent escaping of supercoiling by axial rotations. The model qualitatively agrees with the experimental measurements of supercoiling by means of psoralen photobinding reported earlier [3]. Quantitatively, the levels would reach the levels reported for the sites with low levels of transcription.

**Table 1.** Model setup of bead interactions for chromatin fibre and cohesin. The table shows the interactions divided to bonded and non-bonded. The bonded interactions include bond stretching and angle bending. In the case of the torsionally constrained chromatin fibre additional bonded interaction is used to create penalty for twisting the fibre in terms of dihedral bond between phantom beads (red) attached periaxially to the main chain formed from real beads (blue). The particular interaction is highlighted in green and displayed with corresponding model equation of the interaction and parameter settings. The excluded volume interaction between cohesin beads and chromatin fibre is the same as for the real beads. Additionally, the setting of hydrodynamic drag parameter γ_R_ = 1 is everywhere the same except the position of the bead where it is increased to multiples of 2, 20 and 200 γ_R_.

| **Chromatin Fibre** | | | | | |  |
| --- | --- | --- | --- | --- | --- | --- |
|  | **Interaction** | | **Display** | **Model Equation** | **Parameters** | |
| Bonded | real | Bond stretching | 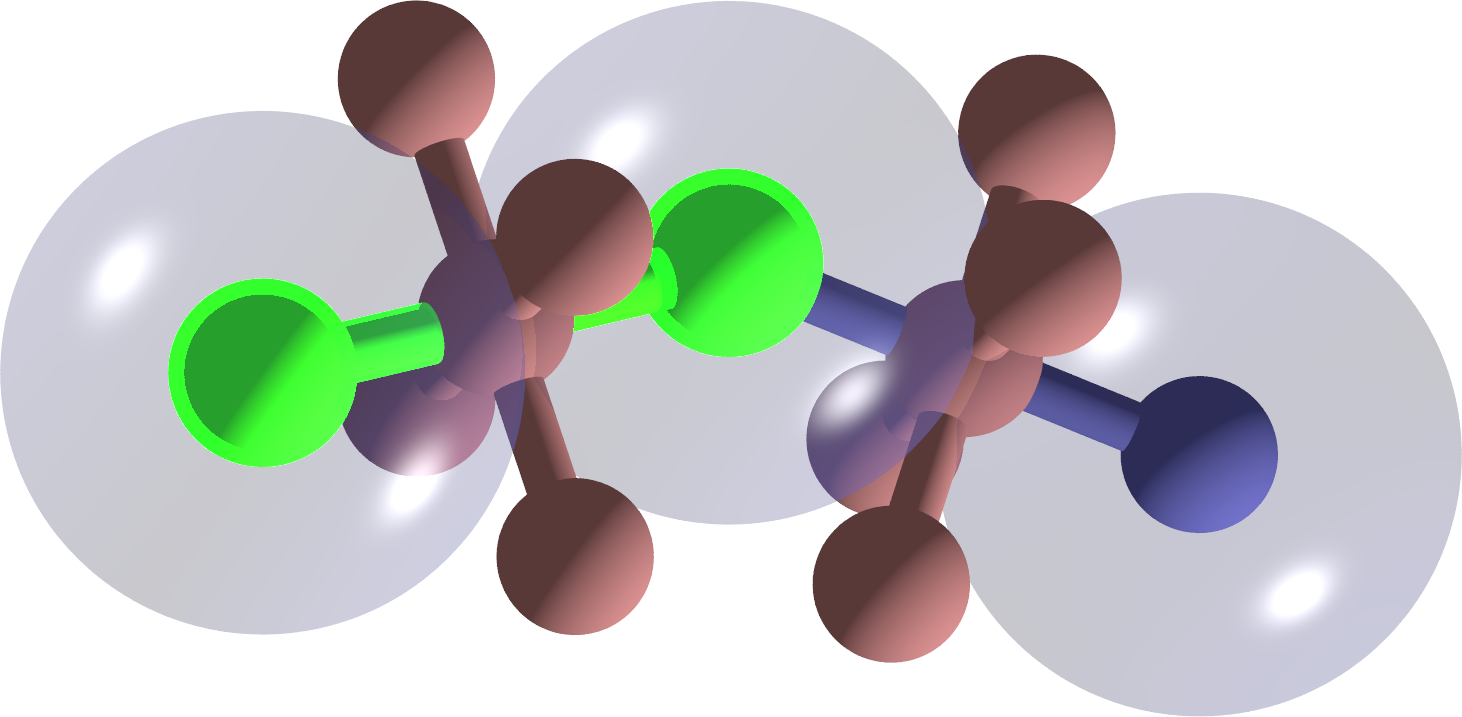 | *V*(*r*) = k (*r* − *r*_0_)^2^ | k = 100*ε* | |
|  |  |  |  |  | *r*_0_ = σ_LJ_ | |
|  |  | Angle bending | 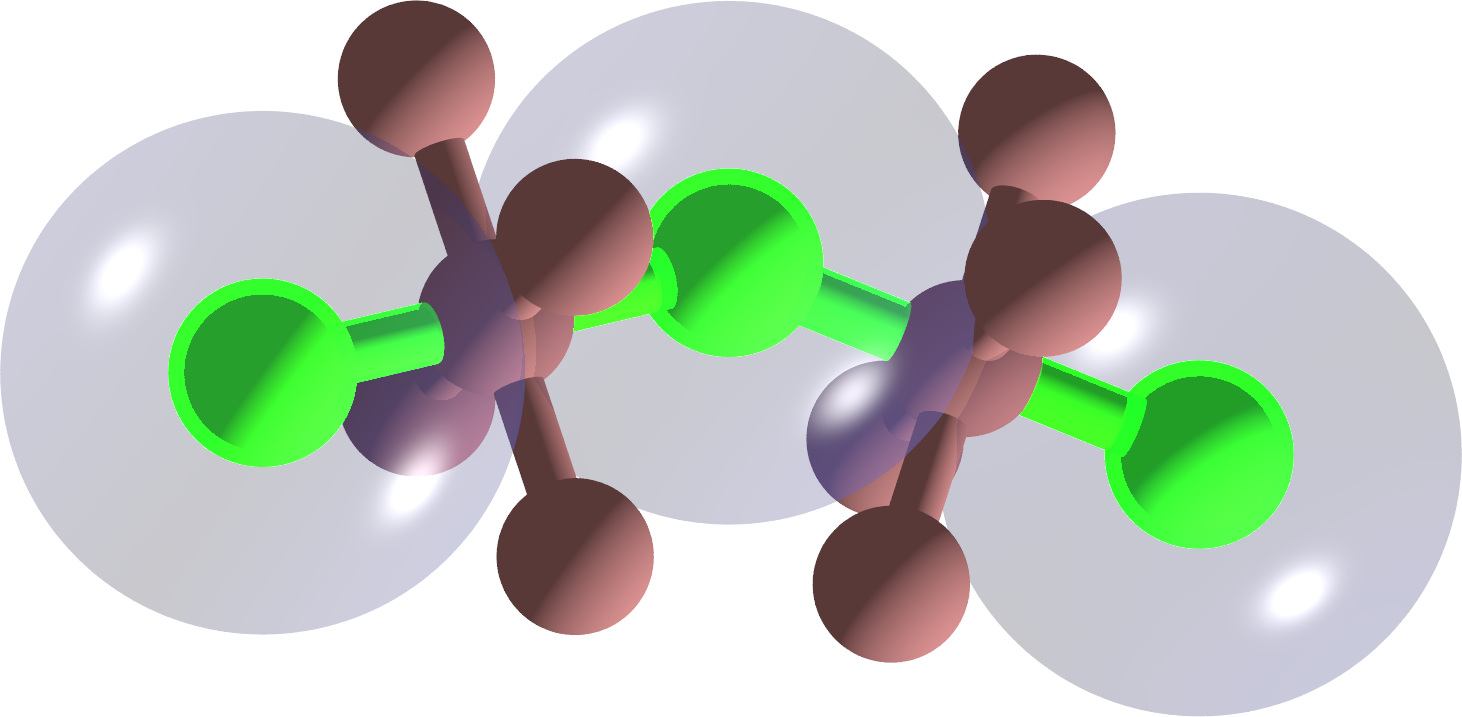 | *V*(*θ*) = 0.5k_b_ (*θ* − *θ*_0_)^2^ | k_b_ = 5*ε* | |
|  |  |  |  |  | *θ*_0_ = π | |
|  | phantom | Bond stretching | 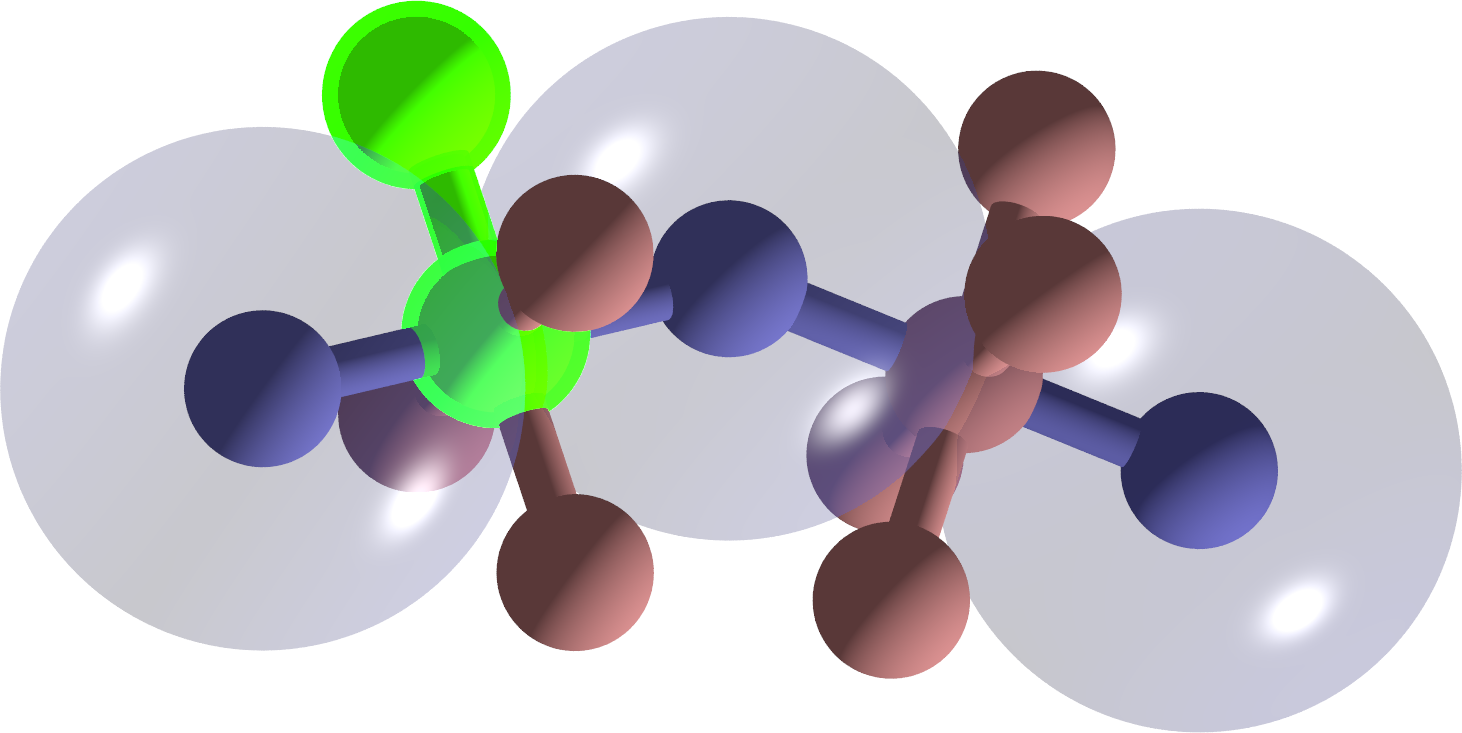 | *V*(*r*) = k (*r* − *r*_0_)^2^ | k = 100*ε* | |
|  |  |  |  |  | *r*_0_ = σ_LJ_ | |
|  |  | Angle bending | 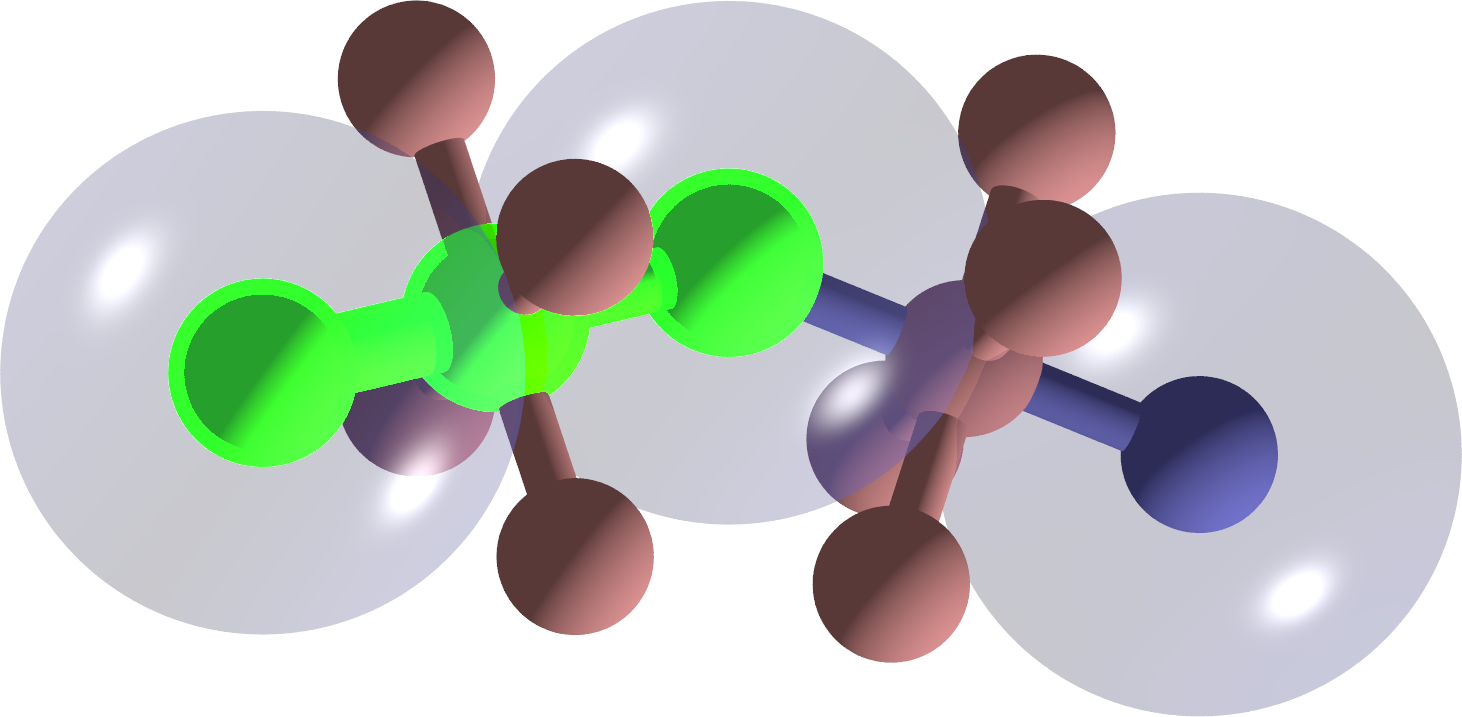 | *V*(*θ*) = 0.5k_b_ (*θ* − *θ*_0_)^2^ | k_b_ =100*ε* | |
|  |  |  | 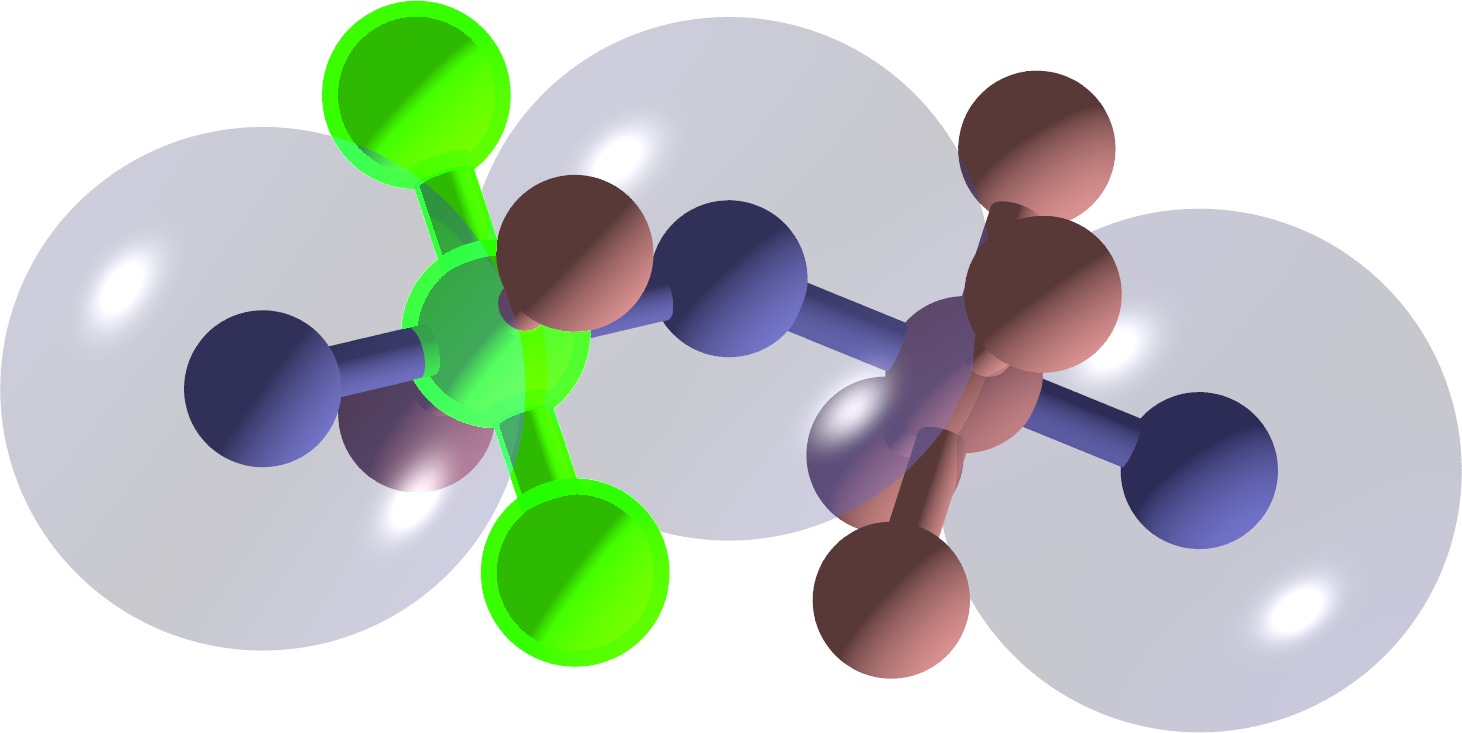 |  | *θ*_0_ = π | |
|  |  |  | 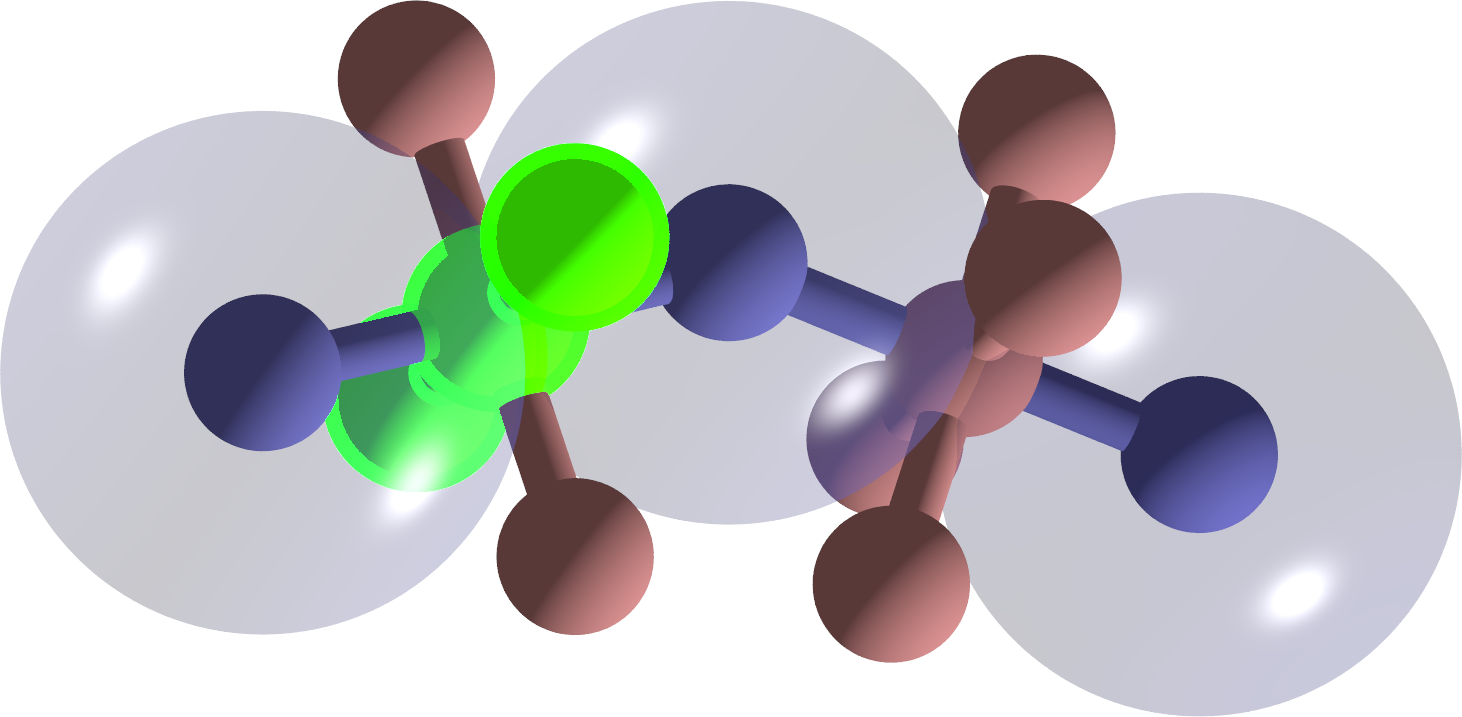 |  | k_b_ =100*ε* | |
|  |  |  | 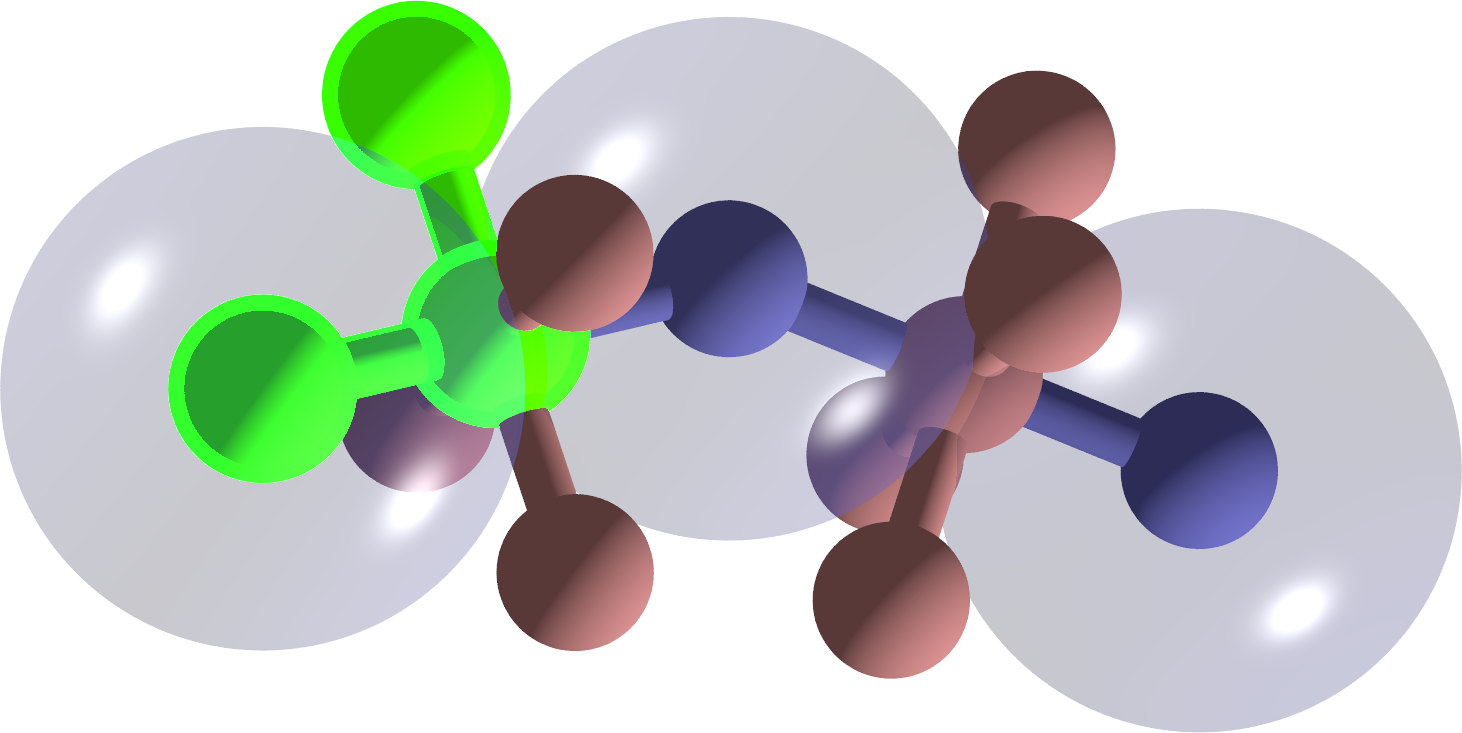 |  | *θ*_0_ = π/2 | |
|  |  |  | 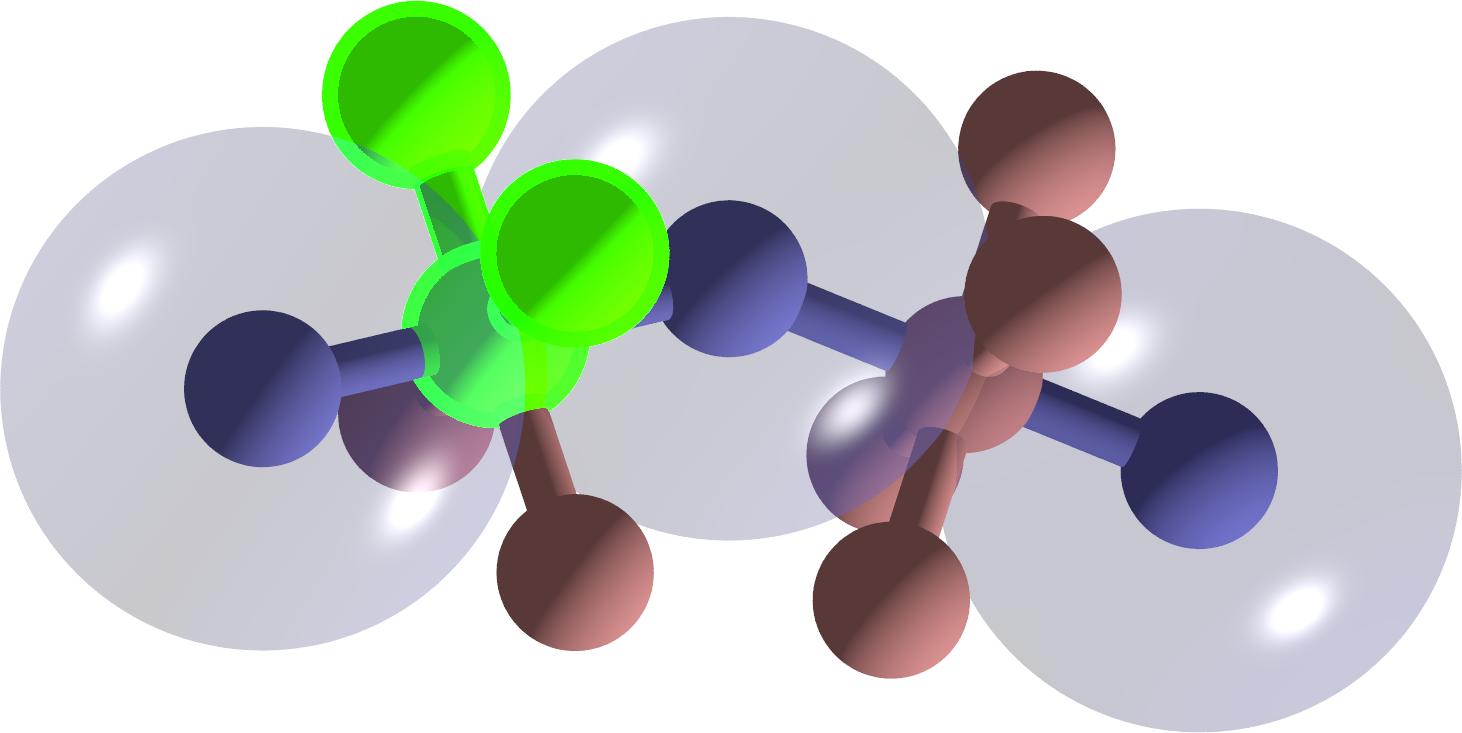 |  |  | |
|  |  | Dihedral | 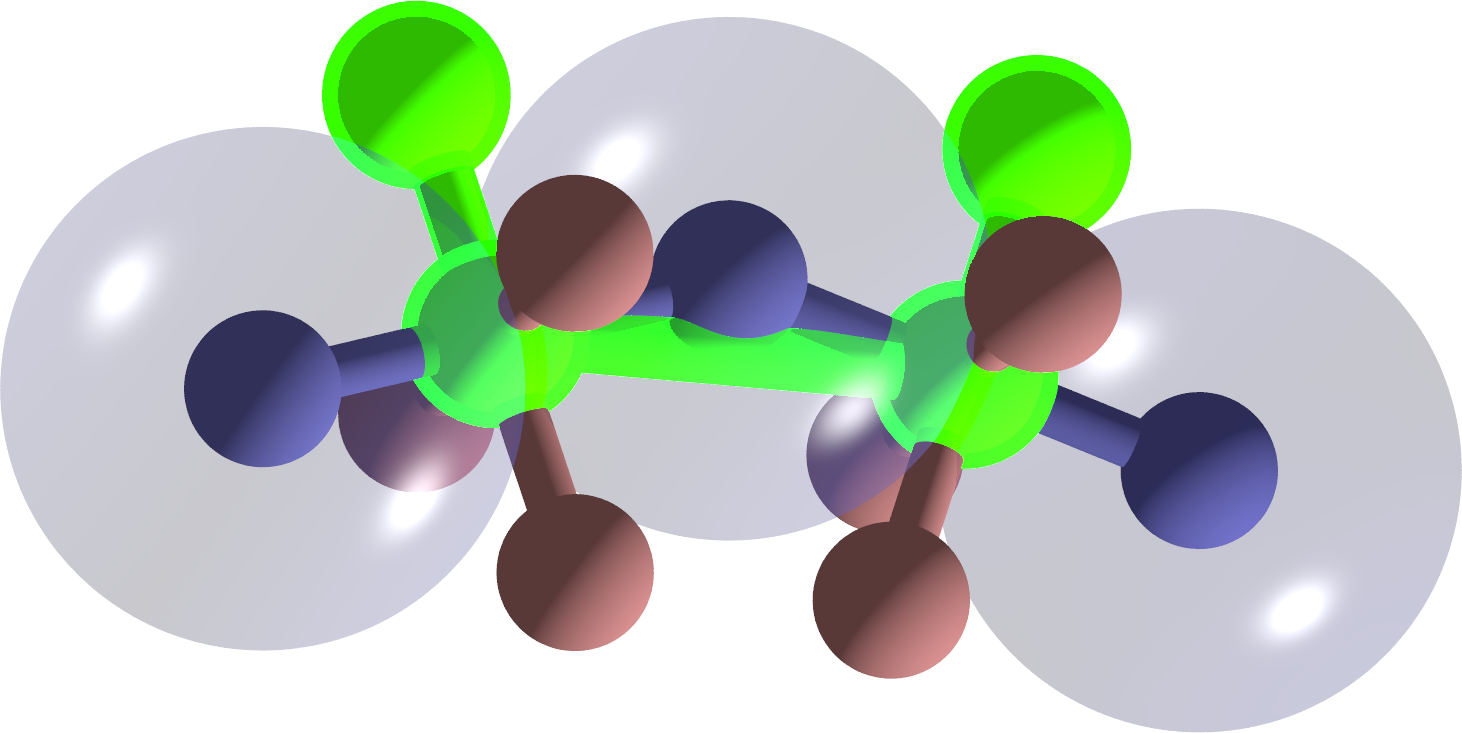 | *V*(*θ*) = 0.5k_φ_ (*φ* − *φ*_0_)^2^ | Supercoiled: k_φ_ = 10*ε*, *φ*_0_ = 0 | |
|  |  |  |  |  | Nicked: k_φ_ = 0, *φ*_0_ = 0 | |
|  |  |  | 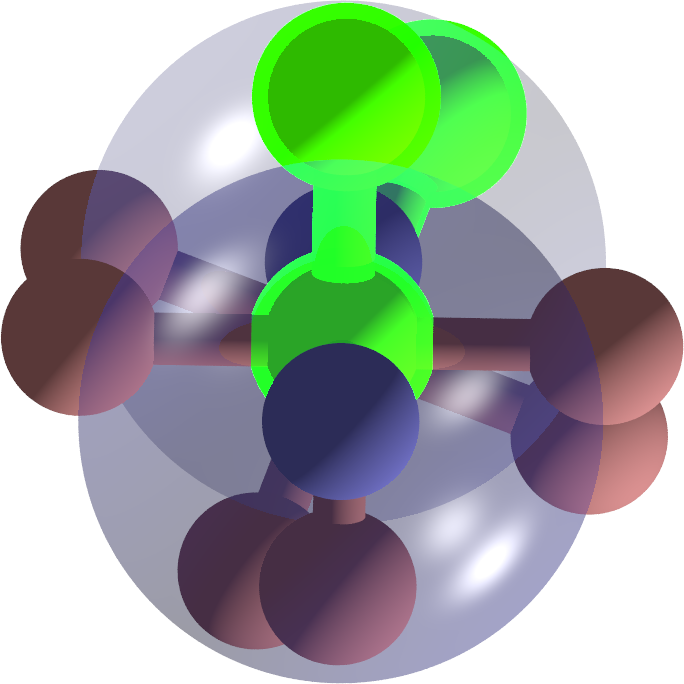 |  | Motor: k_φ_ = 10*ε*, *φ*_0_ = *f*(*τ*) | |
| Non-bond | real | Excluded volume | 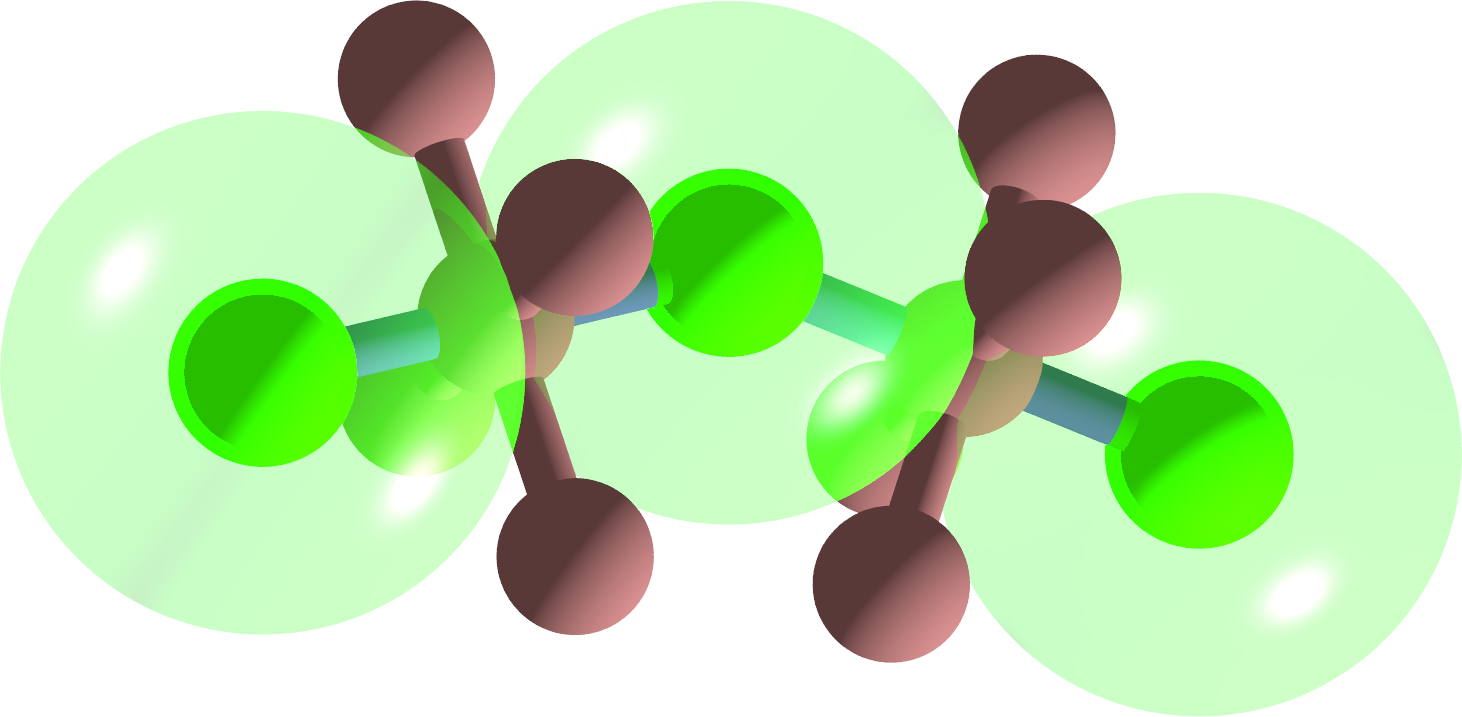 | *V*(*r*) = 4*ε* [(σ_LJ_/r)^12^ − (σ_LJ_/*r*)^6^ + 0.25] | *r* > 2^1/6^: *V*(*r*) = 0 | |
|  |  |  |  |  | *r*_cut_ = 3.0 | |
|  | phantom |  |  | *V*(*r*) = 0 | - | |
| **Cohesin** | | | | | |  |
|  | **Interaction** | | **Display** | **Model equation** | | **Parameters** |
| Bonded | Bond stretching | | 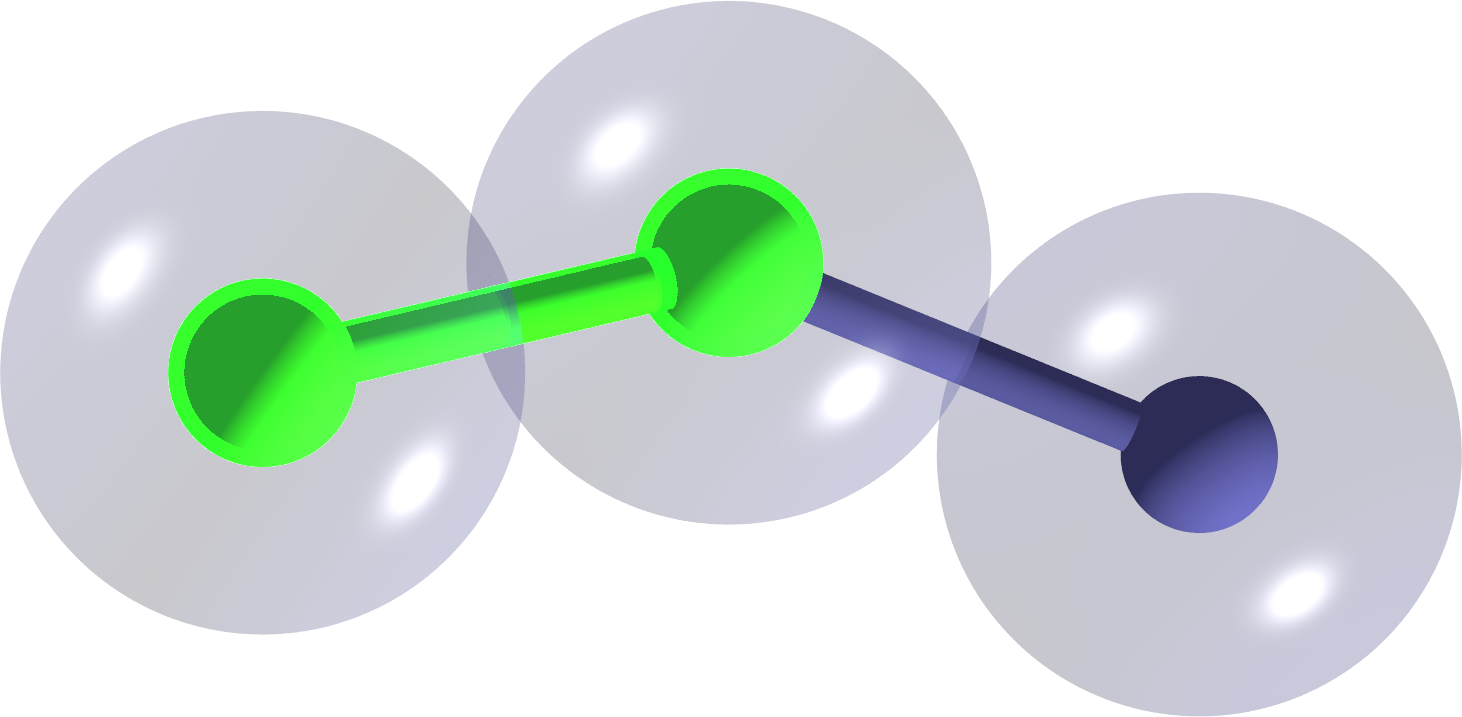 | *V*(*r*) = k (*r* − *r*_0_)^2^ | | k = 100*ε* |
|  |  |  |  |  |  | *r*_0_ = σ_LJ_ |
|  | Angle bending | | 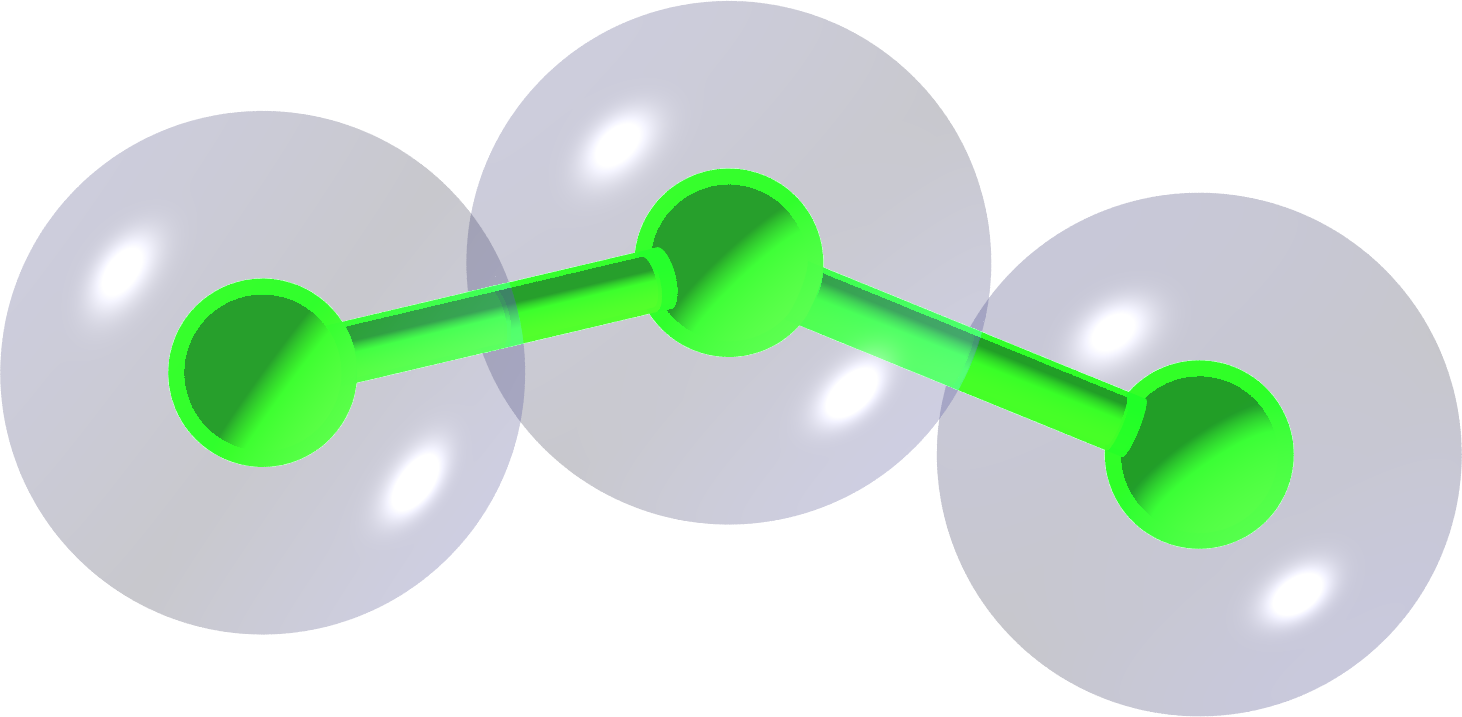 | *V*(*θ*) = 0.5k_b_ (*θ* − *θ*_0_)^2^ | | k_b_ =100*ε* |
|  |  |  |  |  |  | *θ*_0_ = π |
| Non-bond | Excluded volume | | 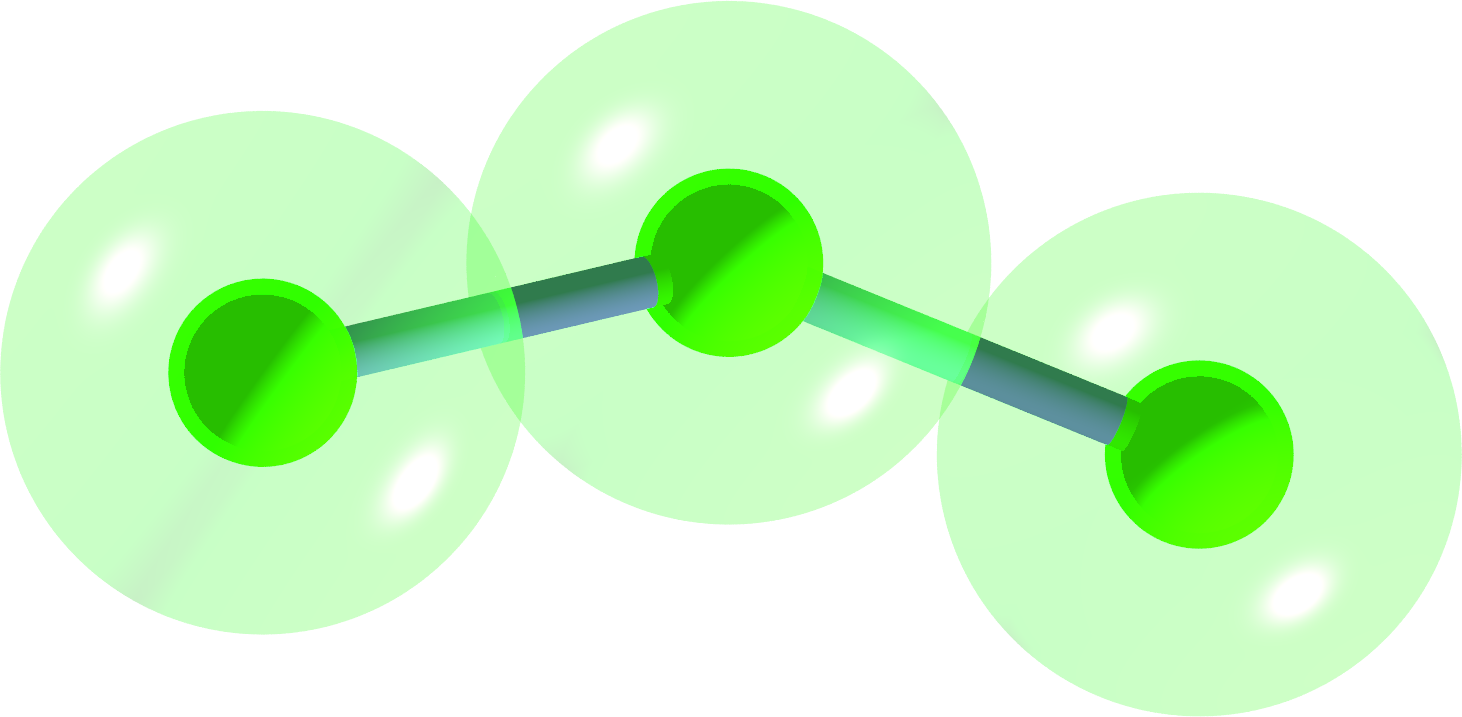 | *V*(*r*) = 4*ε*[(σ_LJ_/*r*)^12^ − (_σLJ_/*r*)^6^ + 0.25] | | *r* > 2^1/6^: *V*(*r*) = 0 |
|  |  |  |  |  |  | *r*_cut_ = 3.0 σ_LJ_ |


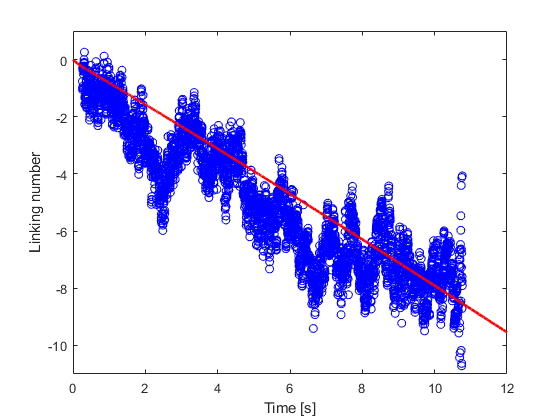

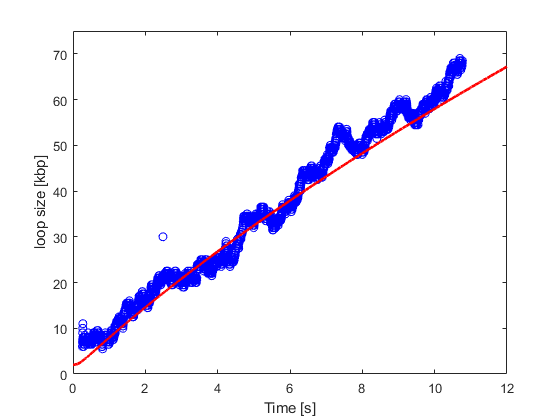


**Figure S1:** Fits of the system of mathematical equations Eq.’s 1 and 2 over the simulated linking number and loop sizes when incorporating the energy terms for stiffness into the energy equation according to Bonato et al [4]. The parameters obtained are *K* = 1550 and *c* = 0.03.


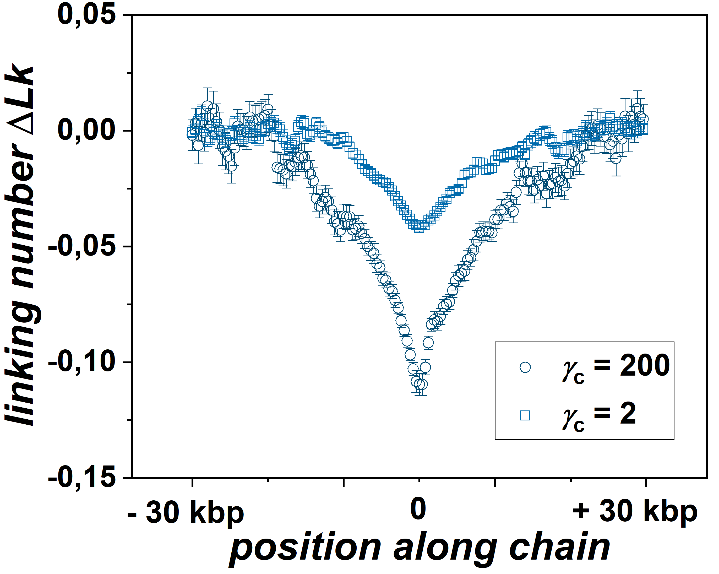


**Figure S2.** The profile of ∆*Lk* obtained as average over the simulation runs along the simulated chromatin fibre with particular setting of the friction between cohesin ring and chromatin fibre γ_c_ = 2 and 200. The higher setting of friction leads to higher accumulations of supercoiling consistently with the reported experimental data by Kouzine et al [3].

Reference:

1. Baranello, L.; Wojtowicz, D.; Cui, K.; Devaiah, B.N.; Chung, H.-J.; Chan-Salis, K.Y.; Guha, R.; Wilson, K.; Zhang, X.; Zhang, H.; et al. RNA Polymerase II Regulates Topoisomerase 1 Activity to Favor Efficient Transcription. *Cell* **2016**, *165*, 357–371, doi:10.1016/j.cell.2016.02.036.
2. Uusküla-Reimand, L.; Hou, H.; Samavarchi-Tehrani, P.; Rudan, M.V.; Liang, M.; Medina-Rivera, A.; Mohammed, H.; Schmidt, D.; Schwalie, P.; Young, E.J.; et al. Topoisomerase II beta interacts with cohesin and CTCF at topological domain borders. Genome Biol. **2016**, *17*, 1–22, doi:10.1186/s13059-016-1043-8.
3. Kouzine, F.; Gupta, A.; Baranello, L.; Wojtowicz, D.; Ben-Aissa, K.; Liu, J.; Przytycka, T.M.; Levens, D. Transcrip-tion-dependent dynamic supercoiling is a short-range genomic force. Nat. Struct. Mol. Biol. **2013**, *20*, 396–403, doi:10.1038/nsmb.2517.
4. Bonato, A.; Brackley, C.A.; Johnson, J.; Michieletto, D.; Marenduzzo, D.; Mareduzzo, D. Chromosome compaction and chromatin stiffness enhance diffusive loop extrusion by slip-link proteins. Soft Matter **2020**, *16*, 2406–2414, doi:10.1039/c9sm01875a.
